# Transactions Alabama Medical Association

**Published:** 1874-10

**Authors:** 


### Bibliographical.

Transactions of the Medical Association of the State of Alabama. Twenty-Seventh Session, 1874. Octavo; paper; pp. 428.

The handsome volume before us does credit to the Medical Association of our sister state of Alabama. The meeting held in April last, at Selma, was, perhaps, the largest and most useful of all the annual sessions of the body, and the volume of Transactions is richly laden with the fruits of the meeting; good and substantial fruits, neatly and fitly garnered for dissemination and future utilization.

Following the journal of daily proceedings, we find the annual address of the president, Dr. George A. Ketchum, of Mobile, largely devoted to the consideration of sanitary medicine. The yellow fever epidemic of last year is treated by Dr. Michel, at Montgomery, and by Dr. Cochran, at Mobile. Dr. Wm. H. Anderson, of Mobile, contributes a report upon dengue. Next follows a paper upon cholera in Birmingham, in 1873, by Dr. M. H. Jordan, and Cholera in Huntsville, by Dr. J. J. Dement. Dr. Jerome Cochran, of Mobile, presents a lengthy paper on the white blood corpuscle in health and disease. Dr. P. Bryce, of Tuscaloosa, follows with the consideration of State aid to hospitals; who advocates a State medical college, supported by the State, and controled by the State medical association.

Dr. T. O. Summers contributes a paper on the contributions of physics and chemistry to practical medicine. Hemorrhagic malarial fever in Alabama is treated by Dr. E. D. McDaniel, of Camden. A paper upon puerperal eclampsia—chloroform—forceps—post-partum hemorrhage, by Dr. F. M. Peterson, follows. He regards eclampsia, as "due to retention in the blood of products of disassimilation, and their poisonous effects upon the nervous system." He relies upon chloroform as "the agent incomparably superior *to all others*." He recommends delivery by forceps, and utterly condemns the lancet.

Dr. J. S. Weatherly, of Montgomery, gives a paper on the anatomy and diseases of the cervix uteri; Dr. W. H. Anderson one on the pneumogastric nerve; and Dr. Benj. H. Riggs a report of the mortuary statistics of the city of Selma.

Dr. E. M. Vasser, of Dallas county, contributes a paper upon the *pycnanthemum linifolium*, or Virginia thyme, or mountain mint, and remarks: "I claim for it a place among the family of botanical remedial agents in atonic dyspepsia. Its action is directly upon the glands of the stomach, causing them to pour out the gastric juice, at once necessary to chymification. * * * Satisfied that this little plant would free the many sufferers from their discomforts and uneasy feelings, and still allow them to indulge in those habits that generated the discomfort, I have given it a fair test, in a number of cases, and with the best results. So well satisfied am I that it is *the* remedy in atonic dyspepsia, when the disease is confined to the gastric glands alone, that I ask for it a place among the family of indigenous remedies, and, as the remedy for dyspepsia, exclaim *Eureka!*"

In the report of the committee on the *contract system* all contracts are discountenanced, except: 1st. When the contract is made with a regular hospital, jail or other public institution, at a price adequate to the labor involved; 2d. When the contract is made with clubs, corporations and societies, for the attendance on the poor members of the same, whom they pledge themselves to assist in sickness and destitution; 3d. It is the sense of this society that while all purely eleemosynary institutions should be attended for a comparatively small professional charge, yet the members of this society should, in common justice of brotherhood and professional interest, make nice distinctions between charity which is real, and that which is only pretended."

Next comes the report of committee on a question of medical ethics, who "Resolved: That the members will refuse professional fellowship and recognition to all regular physicians who may extend any sort of professional service or advice to patients under the care of homœopathic or irregular practitioners—no such service or advice being allowable until the homœopathic or irregular physician has been discharged from all further attendance upon the case."

Then follows Dr. Garonwy Owen with a case of occlusion of the os uteri at term, resulting in delivery through the rectum; Dr. W. D. Bizzell, of Mobile, on aspergillus nigricans; Dr. A. L. Garnett, on thermal waters; Dr. J. S. Bankston, on Jackson county diseases; and Dr. S. D. Seelye, of Montgomery, with the annual oration. The volume closes with a leaf dedicated to the memory of Albert Gallatin Mabry, M.D., ex-President of the Association, who died in Selma, on the 23d February, 1874.

